# Bayesian estimation of physiological parameters governing a dynamic two‐compartment model of exhaled nitric oxide

**DOI:** 10.14814/phy2.13276

**Published:** 2017-08-03

**Authors:** Patrick Muchmore, Edward B. Rappaport, Sandrah P. Eckel

**Affiliations:** ^1^ Department of Preventive Medicine University of Southern California Los Angeles California

**Keywords:** Bayesian inference, exhaled breath, fe_NO_, mathematical model, parameter estimation

## Abstract

The fractional concentration of nitric oxide in exhaled breath (fe_NO_) is a biomarker of airway inflammation with applications in clinical asthma management and environmental epidemiology. fe_NO_ concentration depends on the expiratory flow rate. Standard fe_NO_ is assessed at 50 mL/sec, but “extended NO analysis” uses fe_NO_ measured at multiple different flow rates to estimate parameters quantifying proximal and distal sources of NO in the lower respiratory tract. Most approaches to modeling multiple flow fe_NO_ assume the concentration of NO throughout the airway has achieved a “steady‐state.” In practice, this assumption demands that subjects maintain sustained flow rate exhalations, during which both fe_NO_ and expiratory flow rate must remain constant, and the fe_NO_ maneuver is summarized by the average fe_NO_ concentration and average flow during a small interval. In this work, we drop the steady‐state assumption in the classic two‐compartment model. Instead, we have developed a new parameter estimation approach based on measuring and adjusting for a continuously varying flow rate over the entire fe_NO_ maneuver. We have developed a Bayesian inference framework for the parameters of the partial differential equation underlying this model. Based on multiple flow fe_NO_ data from the Southern California Children's Health Study, we use observed and simulated NO concentrations to demonstrate that our approach has reasonable computation time and is consistent with existing steady‐state approaches, while our inferences consistently offer greater precision than current methods.

## Introduction

The fractional concentration of nitric oxide in exhaled breath (fe_NO_) is a biomarker of airway inflammation with clinical (e.g., asthma) and research (e.g., environmental epidemiology) applications. Nitric oxide (NO) is produced in endothelial cells in airway tissue. The discovery (Gustafsson et al. 1991) that humans exhale measurable quantities of nitric oxide (NO) was soon followed by the discovery that, for a given subject, the concentration of NO exhaled depends strongly on the exhalation rate (Högman et al. 1997; Silkoff et al. 1997). To control for this effect, guidelines for assessment (ATS 1999; ATS/ERS 2005) and interpretation (Dweik et al. 2011) of the fractional concentration of exhaled NO (fe_NO_) have been developed around a standardized exhalation rate of 50 mL/sec (fe_NO,50_). A significant drawback to this approach is that this flow rate provides information on NO arising primarily from proximal airway wall sources (George 2008).

By measuring fe_NO_ at multiple flow rates, it becomes possible to partition the sources of NO into distinct anatomical subregions (Tsoukias and George 1998; Pietropaoli et al. 1999; Högman et al. 2000; Silkoff and Sylvester 2000). In the widely used two‐compartment model (George et al. 2004), the respiratory system is divided into alveolar and airway compartments, and the contribution to fe_NO_ from each estimated separately. Most approaches to modeling multiple flow fe_NO_ assume the concentration of NO throughout the airway has achieved a “steady‐state.” In practice, this assumption demands that subjects maintain sustained flow rate exhalations, during which both fe_NO_ and expiratory flow rate must remain constant. The fe_NO_ maneuver is then summarized by the average fe_NO_ concentration and average flow over a small interval during which the steady‐state assumption appears reasonable. Subjects may only be tested using multiple flow sampling protocols if they can control their exhalation rate with sufficient precision to achieve constant expiratory flows near the protocol targets. Adequate expiratory control may be difficult even for healthy adults (George et al. 2004) or impossible for other groups, such as young children (Linn et al. 2009). Even with excellent expiratory control, fe_NO_ maneuvers at high flows can often display patterns inconsistent with the steady‐state assumption (Puckett et al. 2010).

To overcome limitations of the steady‐state assumption, the goal of this paper is to drop this assumption within the context of a two‐compartment model with a cylindrical airway. Instead, we have developed a new estimation approach based on measuring, and adjusting for, a continuously varying flow rate over the entire fe_NO_ maneuver. Our methodology is similar to the “backward integration” approach introduced in Tsoukias et al. (2001) to analyze samples based on a single breath, although we make fewer simplifying assumptions and explicitly account for the effect of axial diffusion. Because we make no a priori assumptions about the flow rate, there is no inherent need for subjects to control their breathing. Our approach uses measured flow data to continuously adjust the model; therefore, we can analyze data gathered at continuously varying flow rates. This offers the potential for our approach to enable extended fe_NO_ testing with subjects unable to perform existing multiple flow protocols.

In this study, we present the dynamic two compartment model and the Bayesian inference framework we developed to estimate the parameters of the partial differential equation underlying this model. Our approach was originally motivated by mutltiple flow fe_NO_ data from the Southern California Children's Health study. Using existing data from this study, we create a simulated sample of randomly selected subjects to compare our dynamic approach with existing steady‐state approaches. We show that our inference method yields parameter estimates with both superior accuracy and precision compared with existing steady‐state methods applied to the same data. We also show examples of estimates generated using real data, and we end by discussing potential simplifications to the sampling protocol our method may enable.

## Glossary

- *r* and *l* are the airway radius and length (respectively), in cm.
- *z*_0_, *z*_mouth_, and *z*_alv_ are the locations of the sensor, mouth, and alveolar boundary, in cm from *z*_0_.
- *c*(*z*,*t*) is a solution of equation 1, indicating the NO concentration at position *z* and time *t*, in ppb.
- *t*_0_,*t*_1_, … ,*t*_*n*_ are the *n*+1 discrete measurement times, where exhalation begins at *t*_0_ and ends *t*_*n*_.
- *c*_*i*_ :=*c*(*z*_0_,*t*_*i*_) is the model solution at the sensor *z*_0_, ${\hat{c}}_{i}$ is a numerical approximation of *c*_*i*_, and ${\tilde{c}}_{i}$ is the measured concentration, all in ppb and at time *t*_*i*_.
- *v*(*t*) is the linear flow rate, in cm/s.
- *d* is the diffusivity of NO in air, in cm^2^/s.
- *p* is the permeability of airway wall tissue to NO, in cm/s.
- *c*_*w*_ is the concentration of NO in the airway wall tissue in ppb, which is assumed to be constant.

The parameters described in the steady‐state modeling review (George et al. 2004) can be related to the dynamic model as follows:

- fe_NO_  =  *c*(*z*_0_,*t*). The concentration of NO exhaled (also denoted ce_NO_) corresponds to the concentration at the sensor (*z*_0_), and it is the only parameter that can be measured directly. In the dynamic model this varies with time, so it is also a function of *t*.
- ca_NO_  =  *c*(*z*_alv_,*t*). The gas phase alveolar concentration corresponds to the concentration at the alveolar boundary (*z*_alv_). In principle this may vary with time, but often it is assumed to be constant on short (seconds‐minutes) time scales.
- Daw_NO_ =  2*πrlp*. The total airway diffusing capacity is equivalent to the product of the airway surface area and the coefficient *p*. It also equals the product of the airway volume and the source term coefficient 2*p*/*r* in equation 1.
- J^′^aw_NO_ =  2*πrlpc*_*w*_. The maximum total flux of NO in the airway is equivalent to the product of the airway surface area and the coefficients *p*,*c*_*w*_. It also equals the product of the airway volume and the source term constant 2*pc*_*w*_/*r* in equation 1.
- Caw_NO_ = *c*_*w*_.

## Methods

**Figure 1:**
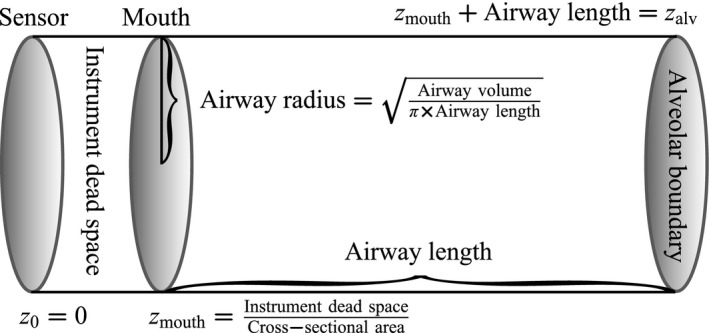
The model airway cylinder. Air flow moves from left‐to‐right (→) during inhalation, and right‐to‐left (←) during exhalation. During exhalation, air is expelled from the alveolar compartment, entering the airway compartment through the right boundary of the model cylinder. To account for dead space in the sampling instrument, the airway cylinder is extended beyond the mouth by the corresponding volume.

Our proposed airway model is a variant of the two‐compartment approach (Tsoukias and George 1998), the primary distinction being we make no assumptions regarding the flow rate. In its simplest form, the two‐compartment airway is assumed to be a cylinder with fixed dimensions (Fig. 1). Unlike the airway compartment, the dimensions of the alveolar compartment may vary. However, at any moment in time the NO concentration is assumed to be constant throughout the alveolar compartment, that is, it is “perfectly mixed.” The original description of the two‐compartment model incorporated a time varying alveolar concentration (Tsoukias and George 1998); in practice it is often assumed to be constant on short (seconds‐minutes) time scales.

The airway cylinder is lined with epithelial tissue containing NO producing cells. The tissue is assumed to be NO permeable, and some of this NO will diffuse from the tissue into the lumen. Exterior to this tissue is bronchial blood, which is assumed to serve as an infinite sink for NO (Tsoukias and George 1998). The tissue NO concentration is assumed to be constant; therefore, depending on the relative NO concentration between incoming air and the airway wall, the airway tissue serves as either an infinite source or sink for airway NO. Although the biological airway ends at the mouth, the cylinder is extended by the instrument dead space volume. In this region, the “airway wall” is assumed to be impermeable to NO; otherwise, it is modeled in the same manner as the rest of the airway. In this regard, the model is equivalent to a cylindrical model with a piecewise constant airway wall permeability.

### The governing equation

The dynamics of NO in the airway are assumed to be governed by the partial differential equation (PDE):

(1) $$\begin{array}{cc} \frac{\partial }{\partial t}c\left(z,t\right)= & -v\left(t\right)\frac{\partial }{\partial z}c\left(z,t\right)+d\frac{\partial^{2}}{\partial z^{2}}c\left(z,t\right)\;\;\\ & +\frac{2p}{r}\left[c_{w}-c\left(z,t\right)\right] \end{array}$$

The three quantities on the right hand side are known as the advection, diffusion, and reaction terms (respectively). In this context the last quantity is also known as a source term, as it represents another source of NO, namely, the airway wall. The contribution from the airway wall is assumed to be proportional to the difference in concentration between the wall and the lumen. More details regarding the physical assumptions underlying the result (eq. 1) can be found in Appendix A.

Our approach to estimation and inference is predicated upon repeated simulation of the underlying physical model. In this framework, “simulating” the model (eq. 1) largely consists of calculating a series of numerical solutions ${\hat{c}}_{0},{\hat{c}}_{1},\ldots ,{\hat{c}}_{n}$, where exhalation begins at *t*_0_ and ends at *t*_*n*_. The method of lines (MOL) technique is applied to the PDE (eq. 1), wherein the spatial (*z*) variable is discretized using finite differences: upwind for the advective term, and centered for the diffusive (see Appendix B for details).

Replacing the spatial derivatives with finite difference approximations yields a large system of ordinary differential equations (ODEs). The time variable remains continuous, and the resultant semi‐discrete problem can be solved numerically when combined with appropriate boundary and initial conditions (discussed in Appendix B). An advantage of this approach is that “off‐the‐shelf” routines designed for arbitrary systems of ODEs can be used to perform the integration (Hundsdorfer and Verwer 2003; LeVeque 2007). Calculating the solution of equation 1 also requires specifying the velocity function *v*(*t*). In a sense, *v*(*t*) “drives” the solution, because it is the only term on the right hand side of equation 1 that varies with time.

### Southern California Children's Health Study data

We demonstrate data processing and model estimation and inference using data from the most recent cohort of the Southern California Children's Health Study (CHS), originally recruited in 2002–2003. Multiple flow fe_NO_ data were collected in March–June 2010 from 1640 children, ages 12–15, in eight of the CHS communities. The study protocol requested that participants perform nine constant flow fe_NO_ maneuvers at four target flow rates: three at 50 mL/sec, and two each at: 30 mL/sec, 100 mL/sec, and 300 mL/sec (Linn et al. 2013).

Samples were collected online using Ecomedics Analyzers (CLD88‐SP with DENOX module), which employ chemiluminescence to measure NO concentrations and ultrasound to measure flow rates. The flow rate measurements are available almost instantaneously, while there is a small delay in the NO signal. This delay is due to a combination of the time required for gas transport between the flow head and the sensor, and a sliding average filter applied to the output signal. The total delay is ∼1 sec, and during testing the analyzer automatically adjusts for this to provide synchronized flow and NO time series output. The NO and flow rate measurements were exported into raw text files at 100  samples/sec. Many of these values are redundant, however, and the effective sampling rates are 50 Hz for flow and 15 Hz for NO. While these data were synchronized at the time of collection, that is not required, and post hoc corrections could also be applied prior to analysis.

### Flow rate data preprocessing

We use a Dormand and Prince (1980) based routine to perform the time integration of equation 1. Like most automatic integration routines, this method adaptively varies the time step size based on running error estimate calculations. Sharp changes in the NO concentration require shorter time steps be taken, dramatically increasing computation time. Because the concentration is flow dependent, sharp changes in the flow rate can precipitate sharp changes in the concentration.

**Figure 2:**
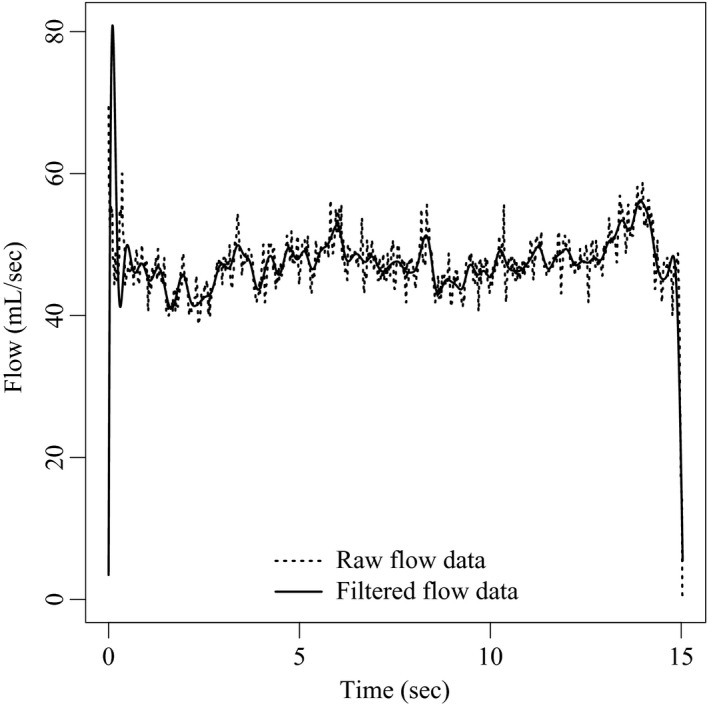
Raw and filtered flow data from a CHS participant's 50 mL/sec maneuver. The raw flow data (dotted line) can oscillate rapidly over a range of 5–10 mL/sec. To dampen these oscillations, the signal is run through a low pass frequency filter with a cutoff of 2.5 Hz. Interpolating the filtered signal defines the estimated flow rate function $\hat{v}\left(t\right)$ (solid line).

Since the solution is calculated at adaptively chosen times, in general it may be necessary to approximate *v*(*t*) at arbitrary values of *t*. Therefore, measurements made at a fixed sampling frequency must be used to estimate the continuous velocity input required by the integration routine. An example of typical flow data for a CHS participant, at the target flow of 50 mL/sec, is shown in Figure 2. Naively interpolating the raw time series can lead to spurious high frequency oscillations in flow rate estimates, mimicking the computational challenges introduced by sharp rate changes.

To dampen these oscillations, the flow rate data is run through a low‐pass frequency filter (Smith 2007). Because the data is analyzed after all of it has been collected, two pass (forward‐backward) filtering is employed via a fourth order Butterworth filter, with a low‐pass frequency threshold of 2.5 Hz. As Figure 2 shows, filtering the signal in this manner retains the gross features, such as the spikes at the beginning, while eliminating the rapid oscillations later on. The estimated flow rate function $\hat{v}\left(t\right)$ is defined by interpolating the filtered signal.

### Model simulation

As illustrated in Figure 1, the position of the sensor is defined to be the origin, *z*_0_ = 0. Therefore, the solution at this point over time corresponds to the model prediction of fe_NO_ measured throughout the maneuver; informally, $\hat{{Fe}_{\mathrm{NO}}}$=${\hat{c}}_{i}$. In addition to the estimated flow rate function $\hat{v}\left(t\right)$, the approximate solutions also depend on the airway parameter values. In these examples, ca_NO_ = 2, J^′^aw_NO_=800, and Daw_NO_ = 5 (values identical to those used in a previous simulation study [Citation Eckel et al. 2014]).

**Figure 3:**
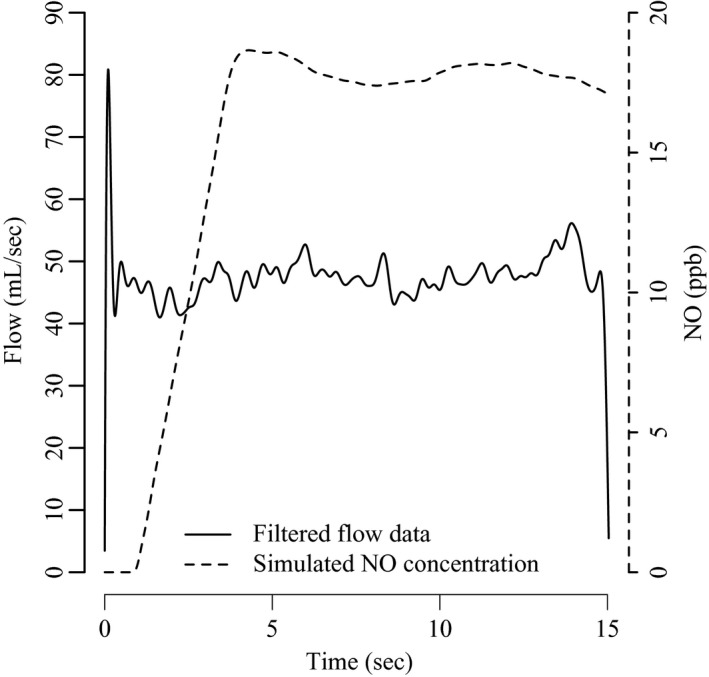
Filtered flow and simulated NO concentration for a CHS participant's 50 mL/sec maneuver. The estimated flow rate function $\hat{v}\left(t\right)$, which interpolates the filtered flow (solid line), can be used to “drive” numerical solutions of the model (eq. 1). At the sensor (*z*_0_), the numerical solution ${\hat{c}}_{i}$ corresponds to an average simulated measurement at time *t*_*i*_. Interpolating the sequence of numerical solutions ${\left\{{\hat{c}}_{i}\right\}}_{i=0}^{n}$ throughout an exhalation (*t*_0_,*t*_*n*_) defines the expected value for fe_NO_ over time (dashed line).

Combining $\hat{v}\left(t\right)$ with the parameters Ca_NO_, J^′^aw_NO_, and Daw_NO_ enables the use of numerical integration to calculate the sequence of approximations ${\hat{c}}_{i}$. The solid line in Figure 3 is the same filtered flow data as shown in Figure 2. The dashed line illustrates the predicted concentration at the sensor throughout the exhalation (synchronized with the flow, so the time scale is shared). The solution ${\hat{c}}_{i}$ is calculated at 100s‐1000s of time steps *t*_*i*_, resulting in a squence of approximations ${\left\{{\hat{c}}_{i}\right\}}_{i=0}^{n}$. Because the integration routine adjusts the step size according to the state of the system, the precise number of steps will vary depending on the input values.

### Multiple flow study simulation

The multiple flow fe_NO_ sampling protocol employed by the CHS was designed to facilitate estimation via existing steady‐state models, and it involved participants performing nine fe_NO_ maneuvers at four target flow rates. Thus, we can validate our model by applying our own method, along with existing estimation techniques, to the simulated data, and then comparing the resultant estimates to the (known) values used during simulation.

Because an exhalation flow profile is such a complicated process, we do not attempt to recreate it via simulation; rather, we use real flow data from the CHS to generate the filtered flow functions $\hat{v}\left(t\right)$ used during simulations. Specifically, we used flow rate data from a stratified random sample of 100 CHS subjects. Sampling strata were determined by fe_NO,50_ level, with 25 subjects selected from each of the categories (in ppb):  <10, 10−25, 25−50, and ≥50. The sample was also restricted to subjects that successfully completed the nine maneuvers specified in the protocol.

**Figure 4:**
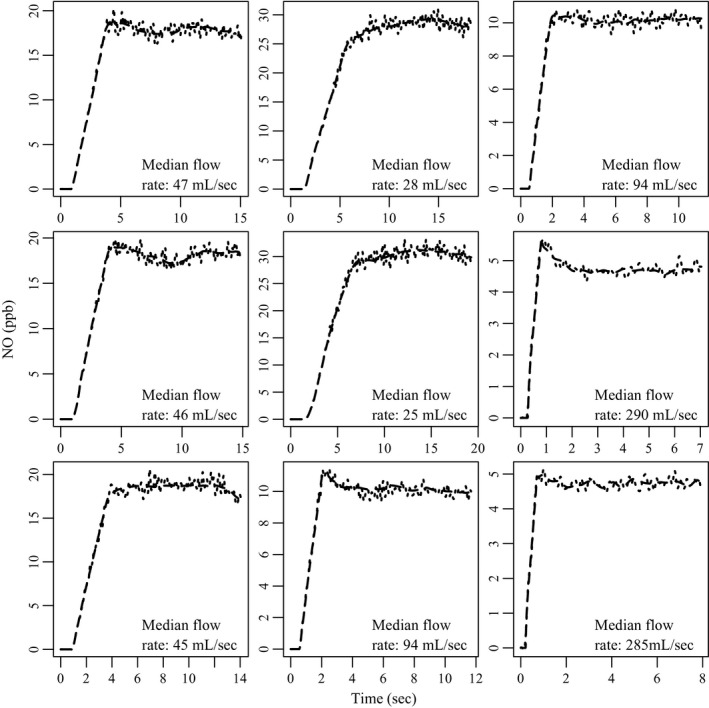
Simulated NO concentrations for nine fe_NO_ maneuvers using observed flow data from 1 CHS study participant. The interpolated sequence of numerical solutions ${\left\{{\hat{c}}_{i}\right\}}_{i=0}^{n}$, which determines expected fe_NO_ over time (dashed line), is a deterministic function of the model parameters. To account for residual sources of variation, the measured values are assumed to be subject to random perturbations which are log‐normally distributed around the deterministic solution. The dashed line in the top left panel corresponds to the dashed line in Figure 3, while the dotted line is the result of simulating observed values ${\tilde{c}}_{i}$ based on ${\hat{c}}_{i}$ in the log‐normal framework described. The process was repeated with the remaining flow data for this subject, yielding nine simulated maneuvers which together satisfy the requirements of the original study (note these are based on average phase 3 plateau flow rates, not the median values shown here).

For every subject, each of their nine maneuvers was used to simulate theoretical NO concentrations *c*_*i*_ as a function of time by combining the observed flow data with the model equation 1 (still using the parameter values ca_NO_=2, J^′^aw_NO_=800, and Daw_NO_=5). The deterministic PDE model leading to the dashed line in Figure 3 is capable of accurately describing many of the qualitative features of exhaled nitric oxide (Shin and George 2002). However, there will inevitably be some deviation between the model prediction *c*_*i*_ and the observed value ${\tilde{c}}_{i}$. To account for this residual variation, we assume $\ln({\tilde{c}}_{i})$ is normally distributed with mean  ln (*c*_*i*_) and variance *σ* ^2^ = 0.1^2^, that is ${\tilde{c}}_{i}$ has a log‐normal distribution. This is the same subject shown in Figure 3 (and Fig. 2), so the dashed line in the top left panel of Figure 4 is identical to the corresponding line in Figure 3. The dotted line is the result of adding independent normal errors to the (log‐transformed) deterministic solution, and the other 8 panels in Figure 4 are the result of repeating this process with the remaining flow profiles for this subject.

### Steady‐state vs dynamic estimation

The most common approaches to estimating ca_NO_, J′aw_NO_, and Daw_NO_, are based on the steady‐state model (George et al. 2004)

(2) $$\begin{array}{cc} {Fe}_{\mathrm{NO}}= & \;\;\frac{J^{\prime }{\text{aw}}_{\mathrm{NO}}}{{\text{Daw}}_{\mathrm{NO}}}+\left({Ca}_{\mathrm{NO}}-\frac{J^{\prime }{\text{aw}}_{\mathrm{NO}}}{{\text{Daw}}_{\mathrm{NO}}}\right)\;\;\text{exp}\left(-\frac{{\text{Daw}}_{\mathrm{NO}}}{\dot{V}}\right) \end{array}$$

relating the parameters of interest to the exhaled concentration ($\dot{V}$ is the volumetric flow rate, which is equivalent to the product *v*(*t*)*πr* ^2^ of the linear flow rate and the cross‐sectional area of the airway cylinder). The model (eq. 2) is a special case of the model (eq. 1), which arises by imposing additional simplifying assumptions (detailed Appendix A).

The direct approach to estimation, introduced in Silkoff et al. (2000), employs a nonlinear regression model by incorporating an additive error term, that is fe_NO_ = ( … )+*ε* (nonLin). A more recent variant, introduced in Eckel et al. (2014), first log transforms the data before performing the nonlinear regression, that is  log (fe_NO_) = log (…)+*ε* (nonLinLog). This method can also constrain parameter estimates to be non‐negative, ensuring consistency with the physical assumptions.

Earlier approaches replaced the exponential function with a polynomial expansion, such as the linear approximations employed by Pietropaoli et al. (1999) in the model ${Fe}_{\mathrm{NO}}={Ca}_{\mathrm{NO}}+J^{\prime }{\text{aw}}_{\mathrm{NO}}/\dot{V}+\epsilon$ (linP), and the model ${Fe}_{\mathrm{NO}}\times \dot{V}=J^{\prime }{\text{aw}}_{\mathrm{NO}}+{Ca}_{\mathrm{NO}}\times \dot{V}+\epsilon$ (linT) described in Tsoukias et al. (1998). A linear function is a poor approximation of the exponential for values near 0, hence higher order polynomials have also been used. The algorithm described in Högman and Meriläinen (2007) (HMA) employs a third order approximation, in addition to ensuring the estimate of Daw_NO_ is positive. While the majority of two‐compartment models employ a cylindrical airway, the model described in Condorelli et al. (2007) (Condorelli) employs a trumpet airway shape, along with a positive diffusion coefficient *d* > 0.

The dynamic approach differs from existing methods by dropping the steady‐state assumption. It is based on estimating the trajectory of fe_NO_ through all phases of exhalation, and there is no requirement for, nor even notion of, fe_NO_ “plateaus.” This is achieved by continuously measuring the flow rate and adjusting for its impact on fe_NO_. Because the estimates generated by the dynamic model are based on a broader spectrum of input values, it has the potential to calculate estimates with greater precision than existing methods. Parameter estimation in this framework is done via Monte Carlo methods, and the point estimates reported here are maximum a posteriori probability (MAP) values generated during Markov chain Monte Carlo (MCMC) sampling, see Appendix C for details (for readers unfamiliar with Bayesian methodology, in this context the reported MAP values are equivalent to maximum likelihood estimates (MLE)).

To asses the uncertainty in point estimates, some methods also produce interval estimates for the parameters of interest. The linP and linT point estimates are based on ordinary least squares (OLS), and confidence intervals can be derived from the assumptions underlying OLS. One of these assumptions is that the residuals are normally distributed. However, when applied to fe_NO_ the true residual distribution is unknown, potentially negating the validity of the intervals. The nonLin and nonLinLog methods employ non‐linear least squares to calculate point estimates, and the associated intervals are based on the asymptotic normality of the maximum likelihood estimator. An advantage of this approach is that it does not require explicit distributional assumptions; however, the results only become exact as *n*→∞. In the context of fe_NO_ sample sizes tend to be small, for example *n* = 9 in the case of CHS subjects, potentially calling into question a large sample approximation. The dynamic model estimates are based on a Bayesian approach, and the associated parameter range estimates are known as credible intervals. These intervals do not require assuming (asymptotic) normality. However, in general they cannot be explicitly calculated, and instead must be estimated with a numerical procedure such as MCMC (as we do here).

## Results

The simulated maneuver‐level data was processed according to the original study protocol, so for each of the 100 simulated subjects, nine estimates of the steady‐state plateau average fe_NO_ concentration and flow rate were generated. Estimates of the parameters ca_NO_, Daw_NO_, and J^′^aw_NO_ were then calculated using two steady‐state approaches (HMA and nonLinLog), along with our novel dynamic approach. The steady‐state methods use the nine estimated plateaus as inputs, while the dynamic approach uses the entire trajectory of each of the nine maneuvers for estimation.

### Simulation study parameter estimates

**Figure 5:**
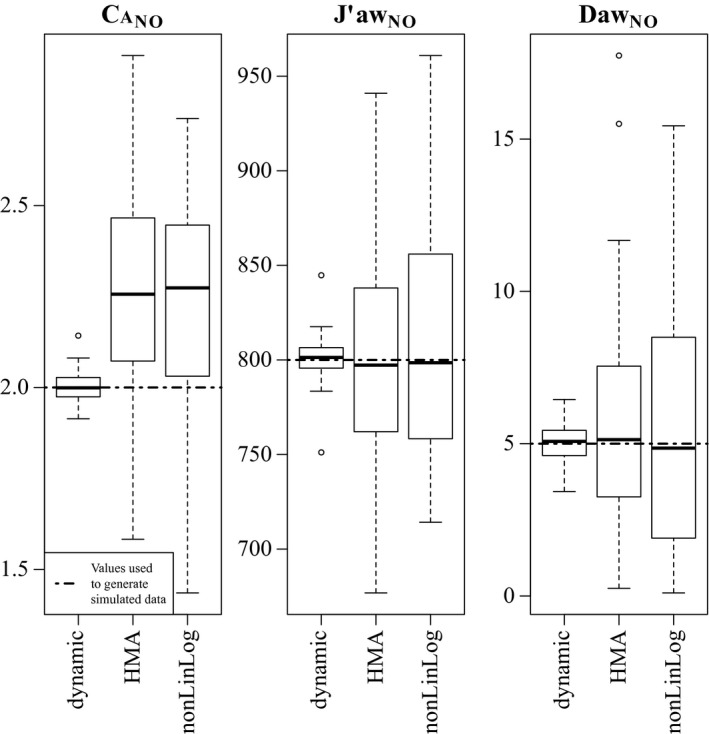
Distribution of NO parameter estimates generated by two steady‐state models, and also our novel dynamic methodology.

The box plots in Figure 5 illustrate the distribution of point estimates generated by all methods for each parameter. The broken horizontal lines indicate the values used to generate the simulated data, hence a “correct” inference would recover these values. For all three parameters the dynamic estimates are centered around the true values, while simultaneously possessing the narrowest sample distribution of any method. The median estimates for J^′^aw_NO_ and Daw_NO_ generated by the HMA and nonLinLog methods also appear very close to the true values, but there is significantly greater variability in the distribution of estimates. The HMA and nonLinLog methods also produced estimates of ca_NO_ that tend to have a positive bias.

To quantify the results illustrated in Figure 5, the mean $\left(\overset{¯}{x}\right)$ and standard error $\left(se(\overset{¯}{x})\right)$ for each sample are shown in Table 1. If we assume the 100 simulated subjects are independent, we can use these estimates to conduct a *t*‐test of equality between the mean of the sampling distribution for $\overset{¯}{x}$, and the known parameter values used during simulation. Because this equality defines an unbiased estimator, rejecting this hypothesis would be evidence of bias in the estimation procedure. The *P*‐*value* columns in Table 1 report the results of these tests using a two‐sided (≠) alternative. For all three parameters, these results support the hypothesis that the dynamic estimates are unbiased. Moreover, it is the only method to provide an unbiased estimate of ca_NO_, and in every case it yields the smallest standard error.

**Table 1:** For each combination of model parameter and estimation method, in the first two columns we report the sample mean and standard error of the 100 simulation study estimates. The third column reports the *P*‐*value* resulting from a test of equality between the mean of the sampling distribution and the true value used during simulation. For these simulated subjects, the dynamic approach is the only one we conclude to be unbiased for ca_NO_, and for all three parameters it provides consistently greater precision than any other method

	ca_NO_=2			J^′^aw_NO_=800			Daw_NO_=5		
	$\overset{¯}{x}$	$se(\overset{¯}{x})$	*P*‐*value*	$\overset{¯}{x}$	$se(\overset{¯}{x})$	*P*‐*value*	$\overset{¯}{x}$	$se(\overset{¯}{x})$	*P*‐*value*
Dynamic	2.00	3.67e‐03	5.96e‐01	801	0.97	4.75e‐01	5.03	5.78e‐02	5.72e‐01
HMA	2.26	2.68e‐02	2.89e‐16	798	5.13	6.84e‐01	5.60	3.17e‐01	6.13e‐02
nonLinLog	2.25	2.67e‐02	6.54e‐15	809	5.76	1.28e‐01	5.34	4.02e‐01	4.03e‐01

The steady‐state assumption requires the time derivative be zero, (∂/∂*c*)*c*(*z*,*t*)=0, reducing the PDE (eq. 1) to a second order ODE. By also assuming the diffusivity of NO in air is zero (*d*=0), the model simplifies further into the first order ODE underlying the equality in equation 2. In a cylindrical airway model, we found the impact of neglecting the diffusion term to be modest, but (statistically) significant.

To demonstrate this, the calculations leading to the dynamic estimates reported in Table 1 were repeated with *d*=0. This produced sample mean estimates for ca_NO_, J^′^aw_NO_, and Daw_NO_ of (2.00, 804, 5.43) (respectively), with corresponding standard errors of (4.71e‐03, 1.30, 0.091). Compared with the true values of (2,5,800), the estimate of ca_NO_ is unaffected (*P*=5.10e‐01), while there is evidence of small but significant positive biases in the mean estimates for both J^′^aw_NO_ (*P*=3.06e‐03) and Daw_NO_ (*P*=8.00e‐06).

### Application to real CHS fe_NO_ data

The plots in Figure 5 and results in Table 1 show our estimation routine reliably recovers known parameter values employed to generate simulated data. To demonstrate our approach applied to real data, we also estimated ca_NO_, J^′^aw_NO_, and Daw_NO_ based on the observed fe_NO_ time series for the CHS subject whose flow samples underlie the simulated profiles in Figure 4.

**Figure 6:**
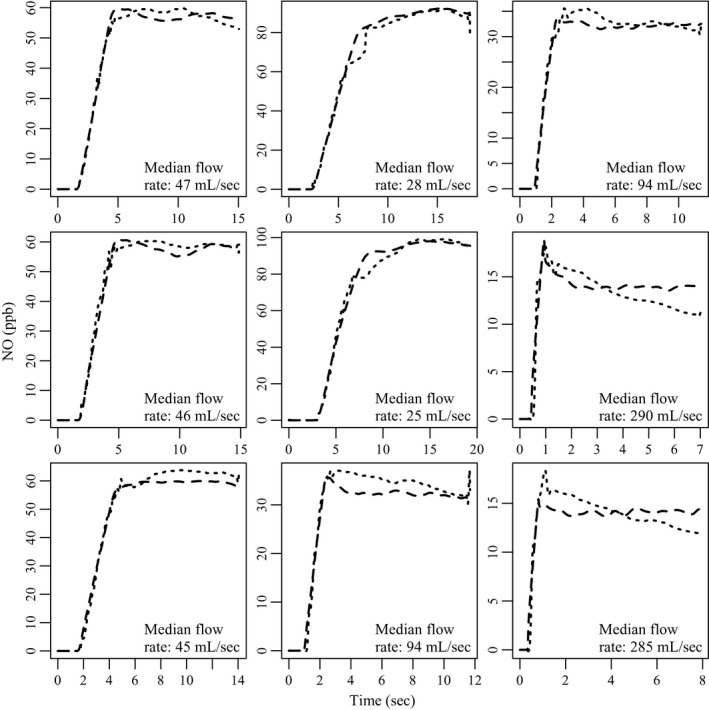
The real (dotted) and simulated (dashed) multiple flow fe_NO_ data for a CHS subject. The simulated profiles are calculated using the MAP parameter estimates generated during MCMC sampling. The flow data used during the simulations illustrated here is identical to what is illustrated in Figure 4, hence any differences between the simulated profiles are due only to the use of different values for ca_NO_, J^′^aw_NO_, and Daw_NO_.

The dotted lines in Figure 6 illustrate the measured fe_NO_ profiles for this subject. While the observed NO profiles have shapes similar to the profiles generated for the simulation study, the fe_NO_ values are significantly (2‐4x) higher in the real data. The dashed lines in Figure 6 illustrate the predicted model solutions based on MAP estimates of the parameter values.^1^ These parameter estimates, which are shown in the first row of Table 2, were calculated using the MCMC sampler described in Appendix C. Plateau NO concentrations and flow rates were also estimated using the real data, and the same steady‐state methods as before were employed to calculate the other point estimates shown in Table 2.

**Table 2:** Parameter estimates based on real fe_NO_ input data. Interval estimates based on frequentist (*conf*) or Bayesian (*cred*) approaches have also been calculated when applicable. The estimated parameter values are significantly larger than the simulation study inputs, although the rank ordering of the estimates largely agrees with the rank ordering in Table 1

	$\hat{{Ca}_{\mathrm{NO}}}$	95% interval	$\hat{J^{\prime }{\text{aw}}_{\mathrm{NO}}}$	95% interval	$\hat{{\text{Daw}}_{\mathrm{NO}}}$	95% interval
dynamic	4.83	(4.52, 5.11) *cred*	2738	(2689, 2795) *cred*	8.52	(7.24, 9.73) *cred*
HMA	6.08	NA	2926	NA	14.0	NA
nonLinLog	6.31	(4.70, 7.92) *conf*	2960	(2552, 3367) *conf*	13.4	(4.96, 21.7) *conf*

While there is no definitive way to asses the accuracy of these estimates, some qualitative features are worth noting. The estimates in Table 2 are significantly (2‐4x) larger than the values used in the simulation study, which is consistent with the fact that this subject has unusually high fe_NO_. As in the simulation study, the HMA and nonLinLog methods produce very similar estimates. Perhaps more interestingly, the rank ordering of the estimates in Table 2 nearly matches the rank ordering of estimates in Table 1.

## Discussion

We have developed a parameter estimation framework for fe_NO_ which treats the observed flow rate as an exogenous input. Multiple flow testing already necessitates measuring the flow rate to ensure protocol compliance; therefore, the flow data we require is, in principle, readily available using current sampling technology. Employing flow data gathered during the CHS, we validated our model by applying current steady‐state methods to 100 subjects in a simulated multiple flow study. We have also developed a novel approach to estimation, and adopting a flow‐adjusted model allows us to use the entire fe_NO_ trajectory for inference. Amongst the simulated subjects and an example CHS participant, this approach significantly improves the precision of flow‐independent parameter estimates.

### Relationship to existing methods

A restriction inherent in a cylindrical airway model is that the cross‐sectional area is fixed. In reality, this area increases with airway generation (Weibel 1963). An alternative airway geometry that incorporates this feature is the “trumpet” model (Shin and George 2002; Condorelli et al. 2007). In the trumpet model, the cross‐sectional area of the airway increases with airway depth; therefore, for the volumetric flow rate to be conserved throughout the airway, the linear flow rate must decrease with airway depth. A cylindrical model might thus be expected to underestimate the significance of the diffusion coefficient *d*, which is corroborated by the finding that axial diffusion plays a larger role in a trumpet versus cylindrical model (Shin and George 2002). This also suggests that the dynamic parameter estimates with *d*=0 may underestimate the true effect of neglecting axial diffusion.

Assumptions regarding *d* are typically made because of computational concerns. The same is true of the assumption that (∂/∂*c*)*c*(*z*,*t*)=0; however, there are significant practical implications to this assumption as well. Because fe_NO_ is flow dependent, subjects must perform sustained exhalations at constant flow rates (i.e., $\dot{V}$ in eq. 2 must be held constant). Official guidelines suggest these plateaus last for at least 3 sec, and that total exhalations last for at least 4 sec in children < 12, and 6 sec for children > 12 and adults (ATS/ERS 2005). Most multiple flow protocols recommend 2–9+ maneuvers be performed (George et al. 2004), and in general performing more maneuvers leads to better parameter estimates. The need to perform repeated, extended exhalations at well controlled flow rates has had a significant negative impact on the potential for routine multiple flow fe_NO_ testing in a clinical setting.

For comparison purposes, the dynamic model estimates are based on the same nine profiles as the steady‐state methods; however, it is not limited to this type of data. By adjusting the model for the measured flow, there is no inherent need for subjects to execute a controlled breathing pattern. Dropping the steady‐state assumption ((∂/∂*c*)*c*(*z*,*t*)=0) potentially has significant practical implications, which is discussed further in the last section.

While the majority of techniques for parameter estimation employ constant flow maneuvers, variable flow approaches have been explored as well, and in Tsoukias et al. (2001) it was shown that all three parameters could be estimated from a single maneuver with a varying flow rate. Similar to our approach, this method uses measured flow data to adjust for the effects of a variable flow rate by performing a backward integration of the flow signal to estimate the airway residence time of an arbitrary bolus sampled at time *t*. Airway NO dynamics are also assumed to be governed by a similar PDE, although the governing equation neglects to account for axial diffusion. Because of this, the model does not precisely predict phases I and II of the profile (e.g., fig. 5 in Tsoukias et al. (2001)), which is a period that should theoretically have high sensitivity to Daw_NO_. By accounting for axial diffusion our approach should be able to better model phases I and II, and by extension could potentially produce more accurate Daw_NO_ estimates. No off‐the‐shelf software is available to implement their approach, and future work should compare/contrast the two methods under a variable flow (or tidal) breathing protocol.

### Limitations

The most significant limitation to our approach is that it is computationally demanding. The estimation routine depends on calculating a numerical solution to equation 1 thousands of times. The single biggest determinant of computation time is the spatial (*z*) resolution required to resolve the dynamics of NO in the airway. Previous studies have found that Δ*z* = 0.2 cm is sufficient (Shin and George 2002); however, our simulations indicate this is inadequate. Specifically, the simulated profiles illustrated in Figure 4 are based on using 1000 grid points (corresponding to a spatial resolution Δ*z*=0.025 cm). We then ran our estimation routine, beginning at 100 grid points, and increasing the number of points until further increases no longer impacted the results. Our experiments show at least 300 points are required, corresponding to a step size of $\Delta z=0.08\overset{¯}{3}$ cm. Using a modern desktop CPU, our estimation procedure runs in 5–10 min per subject.

Our approach to parameter estimation requires detailed time series on both flow and fe_NO_. During the CHS these data were gathered using commercially available analyzers which are capable of recording at 10s (fe_NO_) to 100s (flow) of Hz. However, some newer devices output just a single plateau value (Cristescu et al. 2013; Maniscalco et al. 2016), which is inadequate for fitting our models.

Although the cylindrical two‐compartment model can explain many features of fe_NO_, the airway is not a cylinder, and more sophisticated airway shapes can explain phenomena the standard model cannot. An example would be the steady downward slope observed in the last two maneuvers in Figure 6. One potential explanation for this phenomena is offered by the multi‐compartment, trumpet shaped model introduced in Suresh et al. (2008). In Shelley et al. (2010), this model was shown to be capable of explaining fe_NO_ profiles which decline continuously throughout maneuvers that satisfy official guidelines regarding flow rate stability. Combining our estimation routine with a more realistic airway model could be a natural way to improve the fit of some maneuvers over the standard two‐compartment approach.

### Future directions

While more sophisticated airway shapes can potentially explain trajectories like those in Figure 6, they have typically only been applied to constant flow rate data, and often only in a laboratory setting. The primary appeal of our methodology is that there are no inherent requirements regarding breathing behavior. This potentially opens the door to estimation based on tidal breathing patterns, essentially eliminating the need for subject cooperation during testing. While some preliminary work attempting to use tidal data as a proxy for fe_NO,50_ exists (van Mastrigt et al. 2014), accurately and reliably estimating ca_NO_, J^′^aw_NO_, and Daw_NO_ based on tidal fe_NO_ would be a significant advancement.

From a theoretical perspective, tidal breathing patterns may be preferable to a multiple flow protocol. While current multiple flow protocols typically gather data at 2‐5 different rates, during tidal breathing the observed flow rate continuously ranges over a spectrum of values. Therefore, the dynamic model can potentially be used to estimate the parameters of clinical interest with equal, if not greater precision, by allowing the flow rate to vary continuously. Moreover, this would simultaneously simplify the process, expanding the pool of eligible patients.

## Conflict of Interest

None declared.

## Data Accessibility

## Supporting information




**Appendix S1.** Supplemental information.

## Figures and Tables

**Figure A1:**
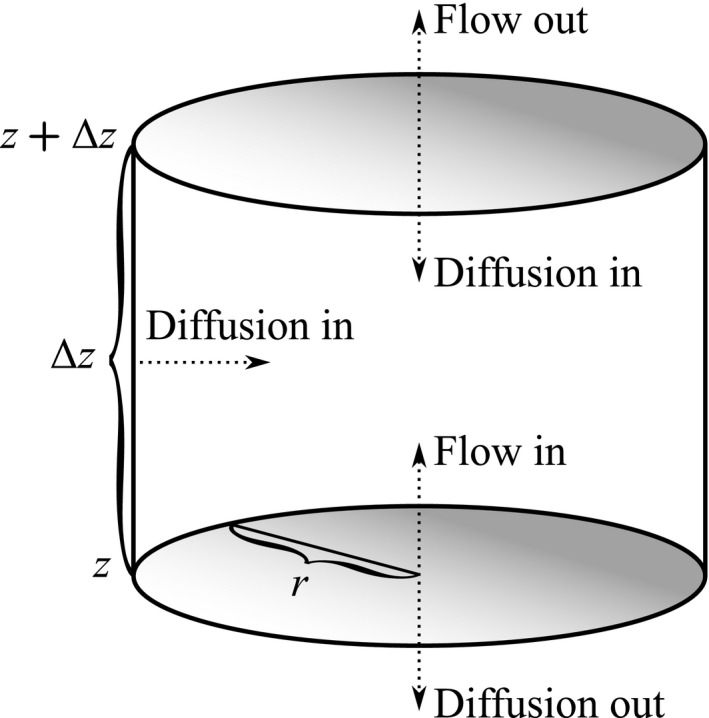
For an arbitrary airway segment of length Δ*z*, and an arbitrary time interval of length Δ*t*, we assume any net change in mass inside the segment must equal the net mass passing through the boundary. As illustrated here, air moves vertically through the airway during exhalation; therefore, the net change due to advection (flow) represents the difference between the mass entering through the lower boundary and exiting through the upper boundary. The airway wall is also assumed to be permeable to NO. Depending on the relative concentrations of the airway wall *c*_*w*_ and the alveolar compartment *c*(*z*_alv_,*t*), it will serve as either a net source or sink of NO. Typically, *c*_*w*_ > *c*(*z*,*t*), yielding a net diffusion of NO from the airway wall to lumen (as pictured here). This also implies that the concentration in the lumen will increase as air moves vertically though the airway, that is, *c*(*z* + Δ*z*,*t*) > *c*(*z*,*t*). This concentration gradient results in net NO diffusion opposite to the direction of flow; therefore, the net change in mass due to diffusion will be the difference between the mass diffusing in through the upper boundary and the mass diffusing out through the lower boundary.

## Note

1 These plots illustrate the model solution over time at one point in space (the NO sensor); however, simulating the model involves calculating the solution over the length of the airway (from the alveolar boundary to the sensor). To illustrate the time evolution of the solution over the entire domain, for each maneuver in Figure 6 an animation illustrating the dynamics of NO throughout the airway has been made available in Appendix S1.

## Appendix A Model description

The airway is assumed to be a cylinder with constant radius *r* and constant length *l* (one or both may be implicitly defined by specifying the airway volume *πr* ^2^ *l* and/or surface area 2*πrl*). The model (eq. 1) results from imposing conservation of (NO) mass throughout this domain. That is, for any time interval (*t*,*t* + Δ*t*), we assume any net change in mass inside an arbitrary region (*z*,*z* + Δ*z*) equals the net mass passing through the regions's boundary over the same time interval.

### Advection of NO axially through the airway

Over the time interval (*t*,*t* + Δ*t*), a cross‐section of air moving at *v*(*t*) cm/s will travel a distance Δ*z*≈*v*(*t*)Δ*t* cm. Therefore, over a time interval of length Δ*t* the mass that flows into the region through the lower boundary is approximately *v*(*t*)Δ*tπr* ^2^ *c*(*z*,*t*). Similarly, the mass that flows out of the region through the upper boundary is approximately *v*(*t*)Δ*tπr* ^2^ *c*(*z* + Δ*z*,*t*), hence the net change in mass due to advection (flow) is approximately −*v*(*t*)[*c*(*z* + Δ*z*,*t*) − *c*(*z*,*t*)]*πr* ^2^Δ*t*

### The airway wall as a source of NO

Depending on the concentration, the tissue lining the airway wall can act as either a source or sink for NO. Typically, we assume the concentration of NO in the airway wall *c*_*w*_ exceeds the alveolar concentration *c*(*z*_*alv*_,*t*), implying the airway tissue serves as a net source of NO. The net transfer of NO from tissue to lumen is assumed to occur at a rate proportional to the concentration difference. Denoting the proportionality constant by *p*, the flux per unit area is *p*[*c*_*w*_ − *c*(*z*,*t*)].Therefore, over a time interval of length Δ*t* the net mass diffusing into the region from the airway wall is approximately equal to the product *p*[*c*_*w*_ − *c*(*z*,*t*)]2*πr*Δ*z*Δ*t*.

### Diffusion of NO axially through the airway

Assuming *c*_*w*_ > *c*(*z*_alv_,*t*) implies that during exhalation the concentration will increase as air moves though the airway, i.e. *c*(*z* + Δ*z*,*t*) > *c*(*z*,*t*). According to Fick's first law, the diffusive flux per unit area will be proportional to the concentration gradient *c*_*z*_(*z*,*t*) := (∂/∂*z*)*c*(*z*,*t*). Denoting by *d* the proportionality constant, over a time interval of length Δ*t* the mass diffusing into the region will be approximately *dc*_*z*_(*z* + Δ*z*)*πr* ^2^Δ*t*. Similarly, the mass diffusing out of the region will be approximately *dc*_*z*_(*z*,*t*)*πr* ^2^Δ*t*, hence the net change in mass due to (axial) diffusion is approximately *d*[*c*_*z*_(*z* + Δ*z*,*t*) − *c*_*z*_(*z*,*t*)]*πr* ^2^Δ*t*.

Imposing conservation of mass requires that over the time interval (*t*,*t* + Δ*t*), the net change inside the region must equal the net mass passing through the boundary. This implies the approximate equality [*c*(*z*,*t* + Δ*t*) − *c*(*z*,*t*)]*πr* ^2^Δ*z* ≈ − *v*(*t*)[*c*(*z* + Δ*z*,*t*) − *c*(*z*,*t*)]*πr* ^2^Δ*t* + *d*[*c*_*z*_(*z* + Δ*z*,*t*) − *c*_*z*_(*z*,*t*)]*πr* ^2^Δ*t* + *p*[*c*_*w*_ − *c*(*z*,*t*)]2*πr*Δ*z*Δ*t*. Dividing both sides by *πr* ^2^Δ*t*Δ*z* then taking Δ*t*,Δ*z*→0 results in the model (eq. 1).

### eq. 2 as a special case of eq. 1

To transform eq. 1 into eq. 2, we begin by assuming the airway NO concentration has achieved a steady‐state, and thus does not vary through time. Mathematically, this is equivalent to assuming (∂/∂*t*)*c*(*z*,*t*) = 0, which also implies *c*(*z*,*t*) = *c*(*z*). Because the concentration is flow dependent, a necessary condition for this to occur is for the velocity to be constant through time, that is *v*(*t*) = *v*. As a further simplification, the diffusivity of NO in air is assumed to be negligible, i.e. *d* = 0, reducing the problem further to a first order ordinary differential equation (ODE).

By substituting (∂/∂*t*)*c*(*z*,*t*) = 0, *v*(*t*) = *v*, and *d* = 0 into equation eq. 1 it simplifies into 0 = −*vc* ^′^(*z*) + (2*p*/*r*)*c*_*w*_−(2*p*/*r*)*c*(*z*). Multiplying through by the airway volume *πr* ^2^ *l*, dividing through by $\dot{V}l$, and rearranging terms yields $c^{\prime }\left(z\right)\;+\;\left[{\text{Daw}}_{\mathrm{NO}}/\left(\dot{V}l\right)\right]c\left(z\right)\;=\;J^{\prime }{\text{aw}}_{\mathrm{NO}}/\left(\dot{V}l\right)$. Generically, this is a linear ODE with constant coefficients of the form *c* ^′^(*z*) + [*a*/*d*]*c*(*z*) = *b*/*d*. Using standard techniques for ODEs, such as separation of variables, the general solution can be shown to be *c*(*z*) = *b*/*a* + *k* exp (−*z*[*a*/*d*]), where *k* is the constant of integration. Therefore, by making the above simplifying assumptions, the general solution of (eq. 1) can be written as $c\left(z\right)\;=\;J^{\prime }{\text{aw}}_{\mathrm{NO}}/{\text{Daw}}_{\mathrm{NO}}\;+\;k\exp\left(-z\left[{\text{Daw}}_{\mathrm{NO}}/\left(\dot{V}l\right)\right]\right)$.

The constant *k* can be found by imposing a boundary condition, and for consistency with Tsoukias and George (1998) the origin (*z*_0_) is shifted from the sensor to the alveolar boundary. That is, by definition the concentration at the origin is equal to the alveolar concentration, or $c\left(0\right)\;=\;Ca_{\mathrm{NO}}$. Combining this condition with the general solution above allows us to solve for the constant, resulting in $k\;=\;Ca_{\mathrm{NO}}\;-\;J^{\prime }{\text{aw}}_{\mathrm{NO}}/{\text{Daw}}_{\mathrm{NO}}$, and hence the particular solution $c\left(z\right)\;=\;J^{\prime }{\text{aw}}_{\mathrm{NO}}/{\text{Daw}}_{\mathrm{NO}}+\left(Ca_{\mathrm{NO}}\;-\;J^{\prime }{\text{aw}}_{\mathrm{NO}}/{\text{Daw}}_{\mathrm{NO}}\right)\exp\left(-z\left[{\text{Daw}}_{\mathrm{NO}}/\left(\dot{V}l\right)\right]\right).$

This solution is applicable along the length of the airway from the alveolar boundary to the mouth, or 0 ≤ *z* ≤*l*. Once air enters the sampling device dead space, both the airway wall concentration and permeability are assumed to be zero. This is equivalent to assuming *c* ^′^(*z*) = 0 in this region, i.e. there is no spatial variation in the concentration. This implies the concentration measured at the sensor equals the concentration measured at the mouth. Mathematically, this means $c\left(l\right)\;=\;Fe_{\mathrm{NO}}$, and inserting *z* = *l* and $c\left(l\right)\;=\;Fe_{\mathrm{NO}}$ into the particular solution yields the equality (eq. 2).

## Appendix B Numerical integration

### Spatial discretizations

To solve the governing PDE (eq. 1) numerically, the spatial (*z*) derivatives are replaced with finite difference approximations based on Taylor series expansions. For the diffusive term, a centered three term Taylor series approximation is employed, (∂^2^/∂*z* ^2^)*c*(*z*,*t*) ≈ [*c*(*z* − Δ*z*,*t*) − 2*c*(*z*,*t*) + *c*(*z* + Δ*z*,*t*)][Δ*z*]^−2^. For the advective term, a biased 4 term Taylor series approximation is employed. The direction of the bias is determined by the direction of flow, as dictated by the sign of *v*(*t*). Specifically, the approximation is oriented with an “upwind” bias; two of the terms in the approximation are chosen on the side from which the flow originates, and only one is chosen from the opposite side.

For example, when *v*(*t*) > 0 the approximation is (∂/∂*z*)*c*(*z*,*t*) ≈ [*c*(*z* − 2Δ*z*,*t*) − 6*c*(*z* − Δ*z*,*t*) + 3*c*(*z*,*t*) + 2*c*(*z* + Δ*z*,*t*)][6Δ*z*]^−1^. An upwind discretization is employed because centered discretizations for advection can lead to spurious oscillations in the numerical approximation. A completely one‐sided discretization can prevent oscillations; however, despite the formal order of the Taylor series, such an approximation will always have first order accuracy (Hundsdorfer and Verwer 2003). By employing a two‐sided, but biased, discretization, higher order accuracy can be achieved while minimizing the potential for oscillatory solutions.

### Boundary and initial conditions

For the PDE (eq. 1) to have a unique solution, initial (time) and inflow boundary (space) conditions must be specified. Moreover, the incoming concentration depends on the direction of flow. During exhalation it corresponds to the alveolar concentration, a parameter to be estimated. During inhalation this concentration is typically the ambient NO level; however, during testing subjects may be provided air that has been “scrubbed” of NO.

By definition, modeling $Fe_{\mathrm{NO}}$ involves modeling exhalation. However, because respiration is cyclic, the terminal condition in one direction of flow becomes the initial condition for the reverse flow. This relationship means that NO measured during exhalation is determined, in part, by the terminal state of the previous inhalation. In principle, the previous inhalation depends, in turn, on the preceding exhalation, which depends on the inhalation before that, continuing ad nauseam.

In practice, higher flow rates diminish this dependence, and at relatively high rates (300+ mL/sec), the terminal airway concentration is effectively independent of the initial. Although 300 mL/sec is a relatively rapid rate for exhalation, it is a relatively slow rate for inhalation. For example, in all 900 maneuvers used for simulation this threshold was cleared every time, typically by factors of at least 2‐3x.

The implication of this phenomena is that calculating accurate estimates of the airway concentration immediately after inhalation does not require knowing the initial airway concentration when inhalation began. The solution can be calculated based on a simple initial condition (i.e. zero everywhere), and the end result will be essentially unchanged. The terminal condition will also depend on the inflow concentration; however, in the case of “scrubbed” air this can be assumed to be zero.

## Appendix C MCMC parameter estimation

We adopt a Bayesian approach to inference (Gelman et al. 2004), and our goal is to characterize the posterior distribution (generically denoted *f*(*θ*|*x*) for parameters *θ* and data *x*). Using Bayes rule, the posterior can be expressed in terms of the likelihood *f*(*x*|*θ*) and a prior distribution *f*(*θ*). For many purposes, including ours, the unnormalized posterior is sufficient, simplifying the relationship between to the posterior, likelihood, and prior to the proportionality *f*(*θ*|*x*) ∝ *f*(*x*|*θ*)*f*(*θ*).

Multiple parameterizations of the model (eq. 1) are possible; for consistency with eq. 2, we work with the vector of parameters $\theta \;=\;(C_{A_{\mathrm{NO}}},J^{\prime }{\text{aw}}_{\mathrm{NO}},{\text{Daw}}_{\mathrm{NO}})$. The data for each subject consists of $x\;=\;\{{\tilde{c}}_{ij}\}$, where ${\tilde{c}}_{ij}$ is the measured concentration at time *t*_*i*_ during maneuver *j* ∈ 1,2, …, 9.

To formulate a likelihood, we assume that by fixing *θ* and solving the corresponding model equation, the model solution can be used to calculate the density of the observed data. If we further assume the observed values ${\tilde{c}}_{ij}$ arise from a shared parametric conditional distribution with density function *f*, and that conditional on the model solutions *c*_*ij*_ the ${\tilde{c}}_{ij}$ are independent, then the likelihood can be written as $f\left(x|\theta \right)\;=\;\prod_{i}\prod_{j}f\left({\tilde{c}}_{ij}|c_{ij}\right)$, where *θ* appears implicitly on the right via the model solution *c*_*ij*_.

When the likelihood *f*(*x*|*θ*) is combined with a prior *f*(*θ*), the (unnormalized) posterior can be easily calculated for any particular set of parameters *θ*. To efficiently explore the posterior distribution we employ a Metropolis‐Hastings style MCMC algorithm (Robert and Casella, 2005), which generically proceeds as follows:

0 Select an initial value *θ* and calculate the likelihood *f*(*θ*|*x*).
1 Propose a new value *θ* ^′^ using a transition kernel *q*(*θ*→*θ* ^′^), and calculate the likelihood $f(\theta^{\prime }|x)$.
2 Accept the proposed value with probability $\min[1,\frac{f\left(x|\theta^{\prime }\right)f\left(\theta^{\prime }\right)q\left(\theta^{\prime }\to \theta \right)}{f\left(x|\theta \right)f\left(\theta \right)q\left(\theta \to \theta^{\prime }\right)}]$.
3 If the proposal is accepted set *θ* = *θ* ^′^, $f\left(\theta |x\right)\;=\;f\left(\theta^{\prime }|x\right)$ then return to 1; otherwise, return to 1.

The choice of transition kernel *q* can have a significant impact on the efficiency of this type of algorithm. Finding an optimal *q* can be difficult; however, there are a number of more recent MCMC algorithms which incorporate an “adaptive” transition distribution (Roberts and Rosenthal 2009). To better account for variability in the posterior across individuals, the adaptive Metropolis algorithm of Haario et al. (2001) is employed to automatically calibrate the proposal distribution against the target. This has the dual benefit of both increasing the efficiency of our sampler, while also simplifying the user experience by largely automating the choice of transition kernel.
