# Supplementary material for: Bayesian estimation of physiological parameters governing a dynamic two‐compartment model of exhaled nitric oxide
Source: Physiol Rep. 2017 Aug 3;5(15):e13276. doi: 10.14814/phy2.13276 (PMC5555880; doi:10.14814/phy2.13276)
Supplement: Supplementary file 1 — Appendix S1. Supplemental information. [file PHY2-5-e13276-s001.zip › supplement.pdf]

Supplemental information

The dotted lines in Fig. 6 indicate the observed $\mathrm{FE_{NO}}$ trajectories for a real CHS participant. Using our novel methodology, the most likely values for $\mathrm{CA_{NO}}$, $\mathrm{J'aw_{NO}}$, and $\mathrm{Daw_{NO}}$ were calculated based on these profiles. With these estimates as inputs, the model was run again, resulting in the dashed lines in Fig. 6. However, the values illustrated represent just a sliver of the overall simulation results.

To illustrate the complete model predictions, we have created a series 9 animated profiles, one for each simulated maneuver. To make some details more apparent, the animations have been slowed to 1/2 speed (the "Elapsed time:" at the bottom of the screen shows the original clock time). The predictions shown by the animations extend the results shown by Fig. 6 in two ways.

First, the figures only illustrate exhalation, while the complete model also estimates the airway concentration throughout the preceding inhalation. Each animated maneuver begins at a negative time, indicating the inhalation phase. During this period, flow is defined to be negative, and its value is indicated by the bar on the left. Compared to exhalation, inhalation occurs at a faster rate, but for a shorter period of time. The inhalation phase ends at time 0, which is defined by the transition from negative to positive flow (this also corresponds to time 0 in the panels of Fig. 6).

The predicted $\mathrm{FE_{NO}}$ profiles in Fig. 6 correspond to the model solution at a single point (the NO sensor) over time. However, calculating the solution at this point requires calculating the solution throughout the length of the airway. Therefore, the second way the animations extend the figures is by using these values to illustrate the predicted concentration over the entire domain. This extends from the alveolar boundary to the NO sensor, providing a way to visualize NO dynamics in otherwise inaccessible regions.

The color scale is customized to each maneuver. It has been calibrated to range from 0 ppb (blue) to the maximum observed concentration (red). The maximum value is largely determined by the target flow rate, so maneuvers targeting different rates are on widely varying color scales. During the exhalation phase, the concentration at the alveolar boundary is fixed at $\mathrm{CA_{NO}} = 4.83$ (from Table 2). During the inhalation phase it is allowed to vary, although it is generally within within 1 or 2 ppb of the $\mathrm{CA_{NO}}$ estimate.
