# Supplementary figures and images for: Bayesian estimation of physiological parameters governing a dynamic two‐compartment model of exhaled nitric oxide
Source: Physiol Rep. 2017 Aug 3;5(15):e13276. doi: 10.14814/phy2.13276 (PMC5555880; doi:10.14814/phy2.13276)

NO (ppb)

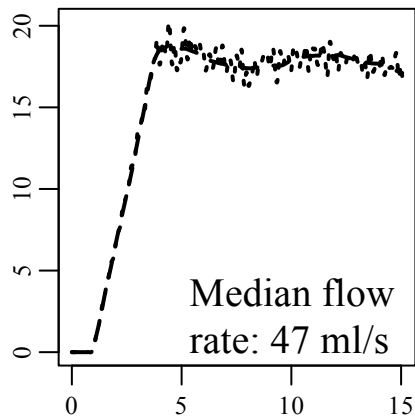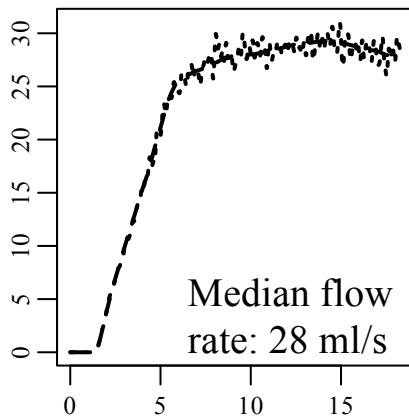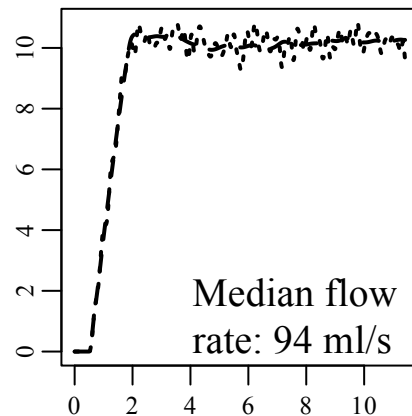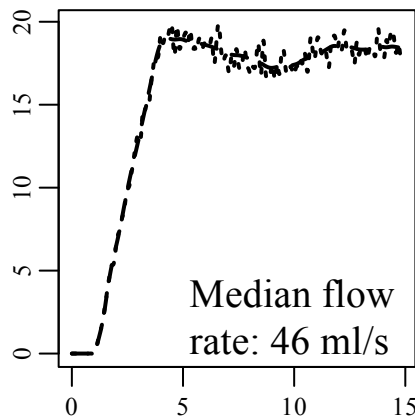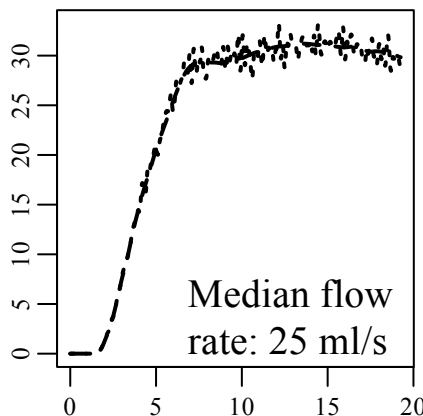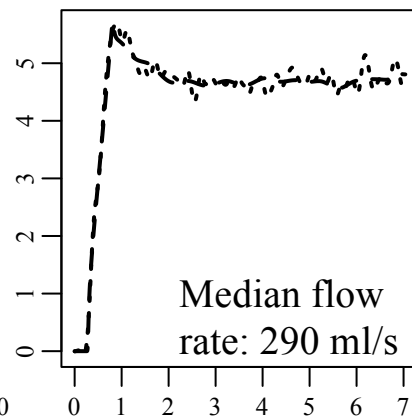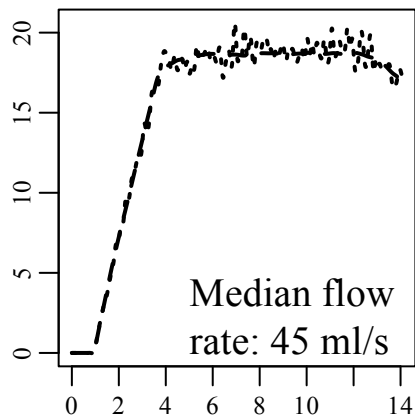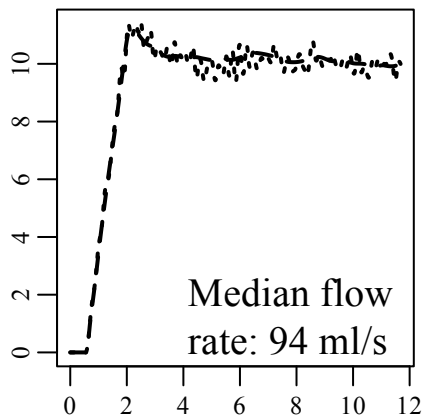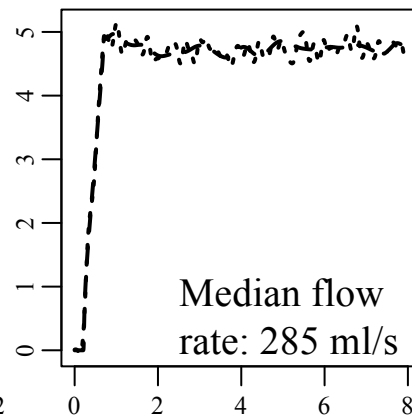

time (s)

Supplement: Supplementary file 1 — Appendix S1. Supplemental information. [file PHY2-5-e13276-s001.zip › SimulatedProtocol.pdf]

**C<sub>AN<sub>O</sub></sub>**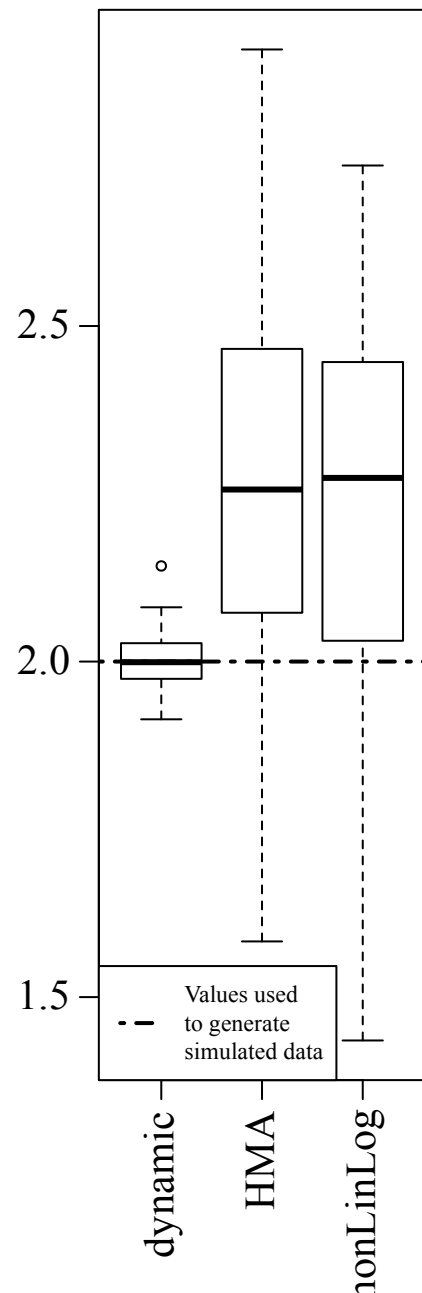**J'<sub>a</sub>W<sub>NO</sub>**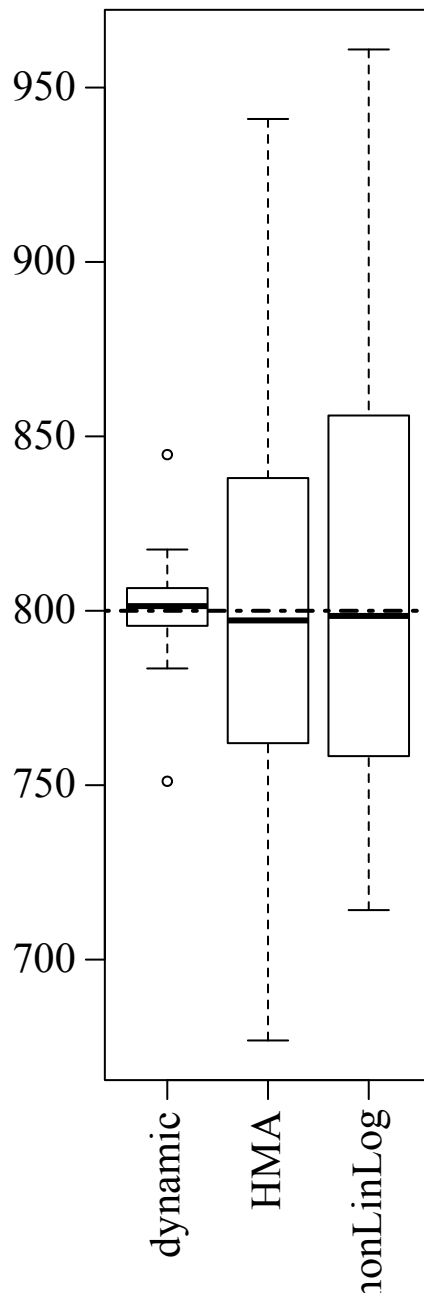**D<sub>a</sub>W<sub>NO</sub>**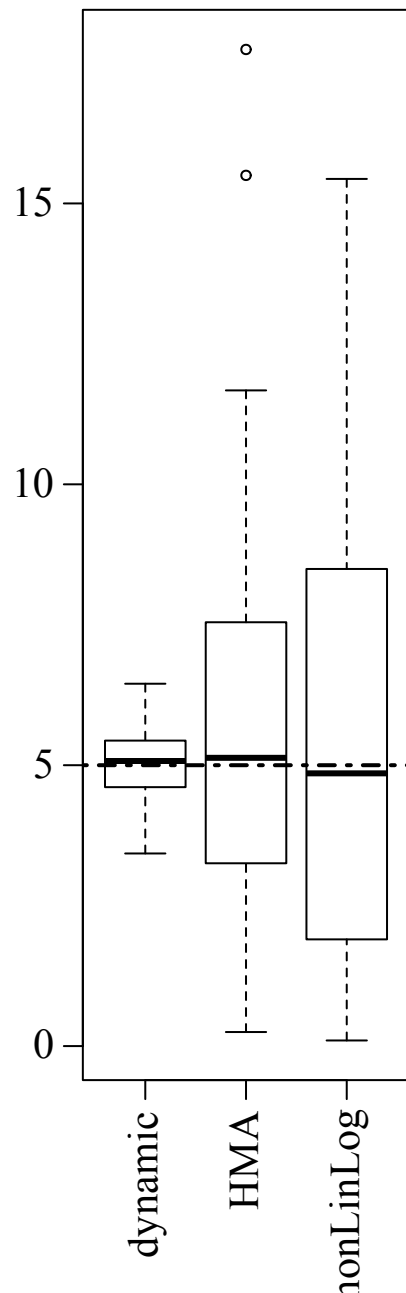

Supplement: Supplementary file 1 — Appendix S1. Supplemental information. [file PHY2-5-e13276-s001.zip › ParameterEstComparison.pdf]

NO (ppb)

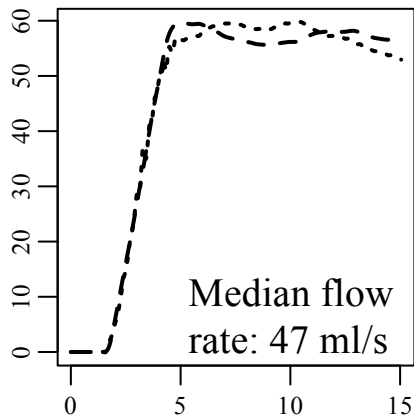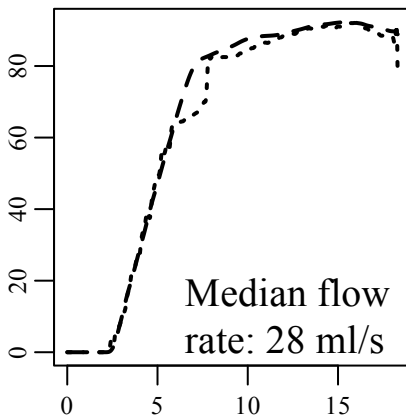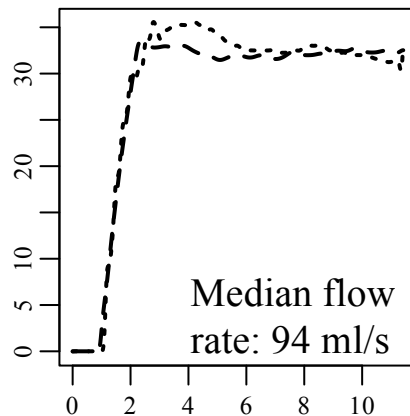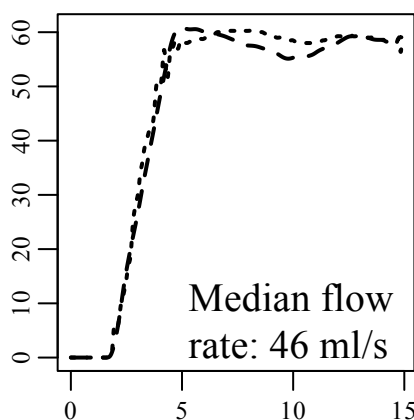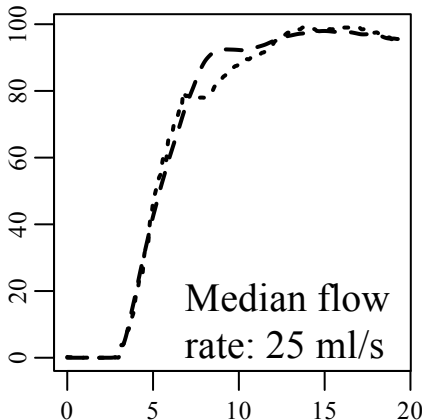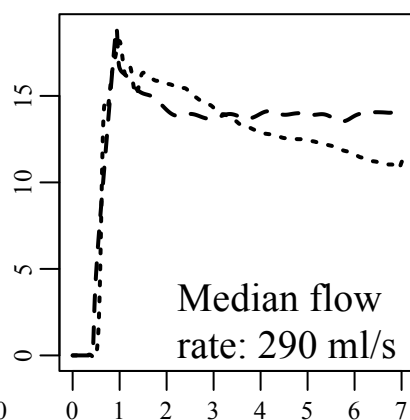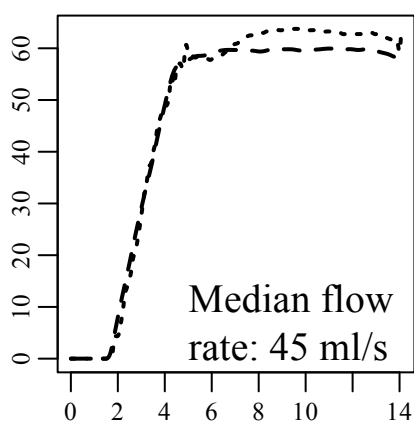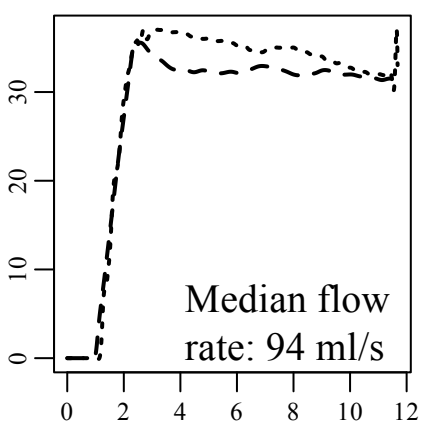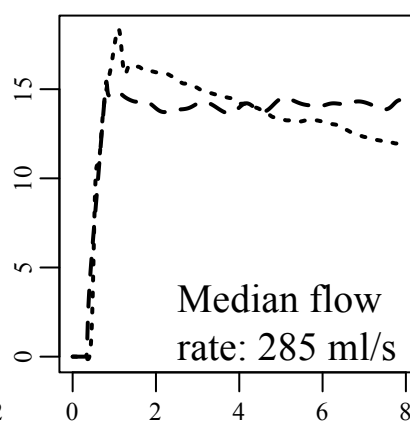

time (s)

Supplement: Supplementary file 1 — Appendix S1. Supplemental information. [file PHY2-5-e13276-s001.zip › PredictedProfiles.pdf]

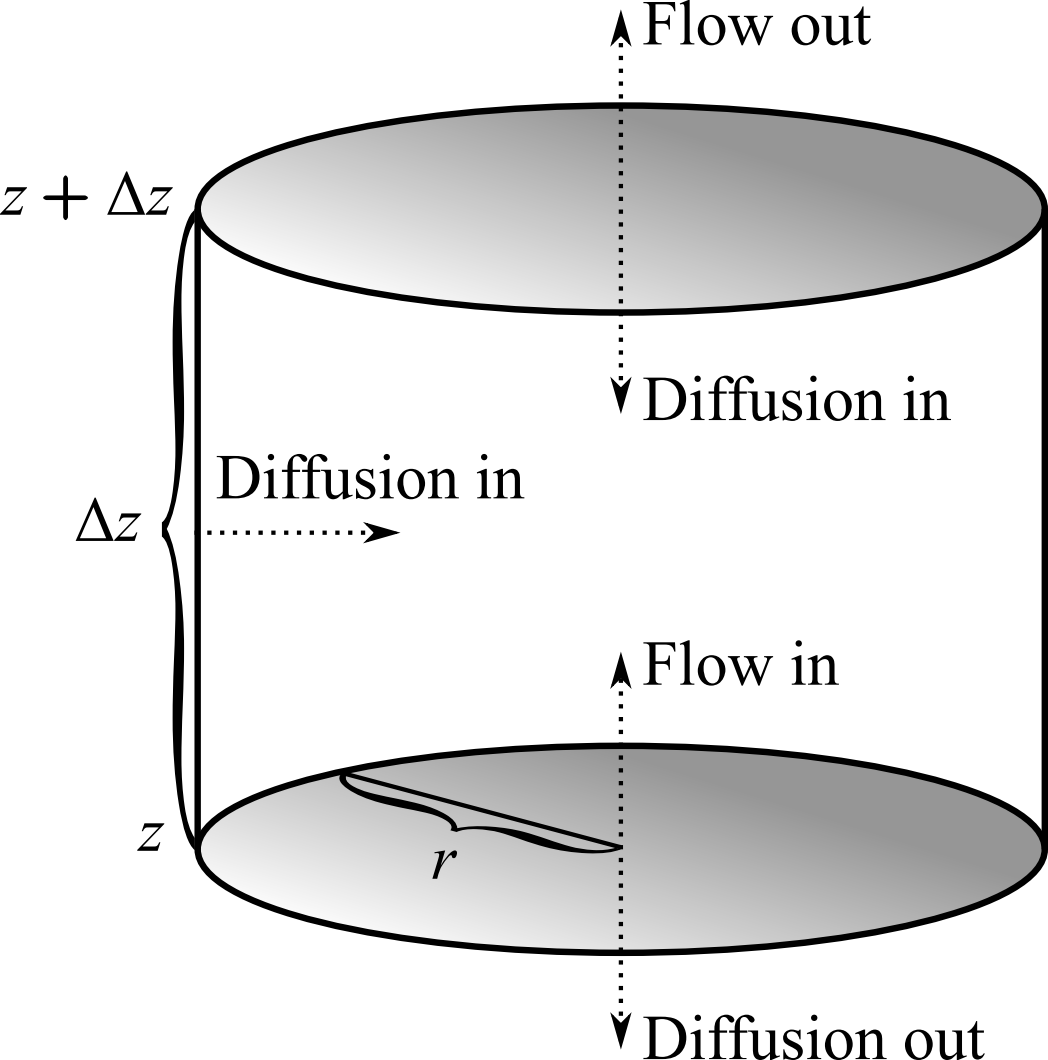

Supplement: Supplementary file 1 — Appendix S1. Supplemental information. [file PHY2-5-e13276-s001.zip › AirwaySlice.pdf]

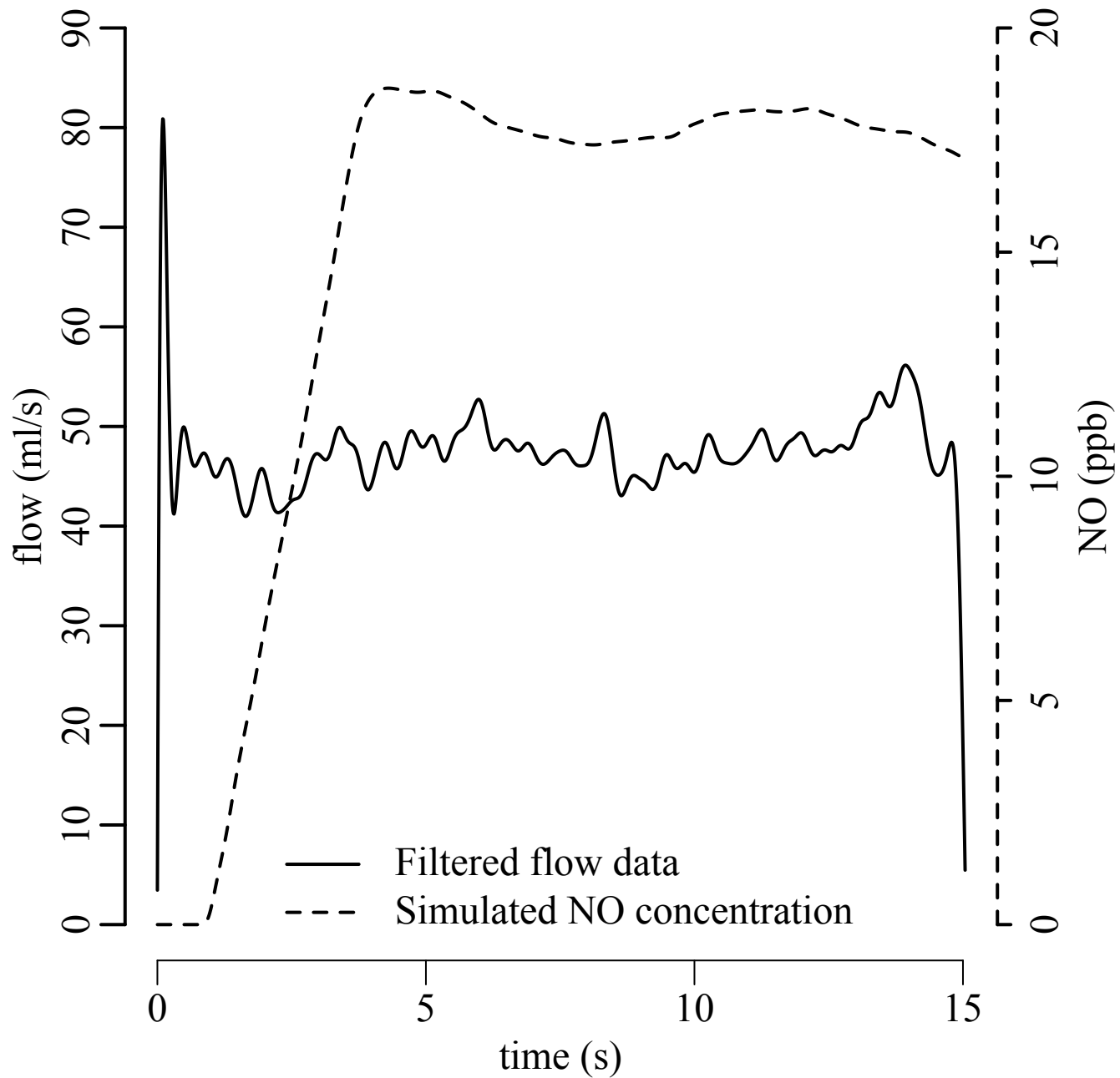

Supplement: Supplementary file 1 — Appendix S1. Supplemental information. [file PHY2-5-e13276-s001.zip › FlowAndENO.pdf]

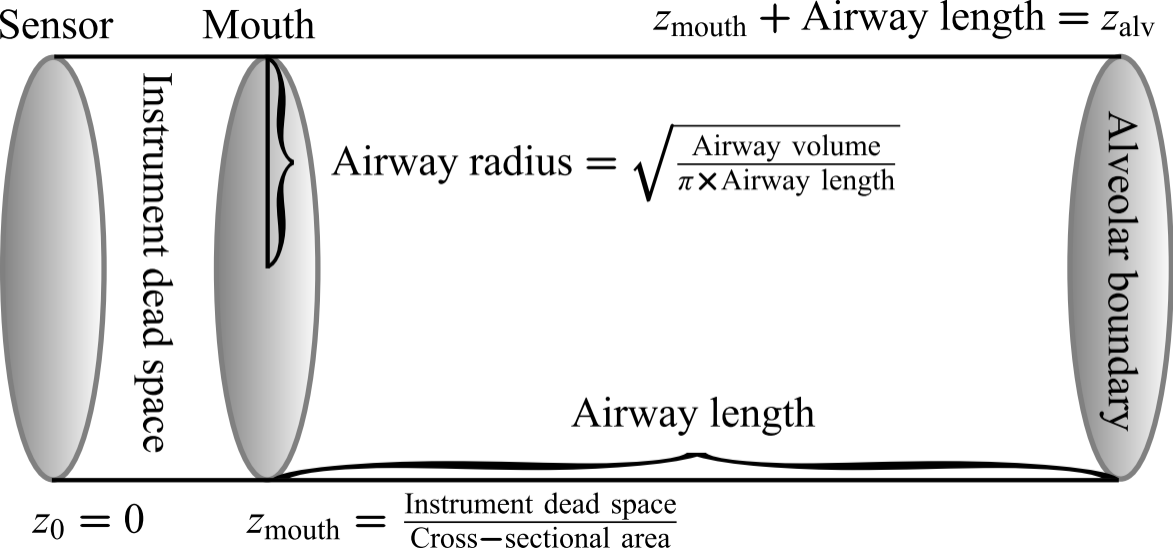

Supplement: Supplementary file 1 — Appendix S1. Supplemental information. [file PHY2-5-e13276-s001.zip › ModelCylinder.pdf]

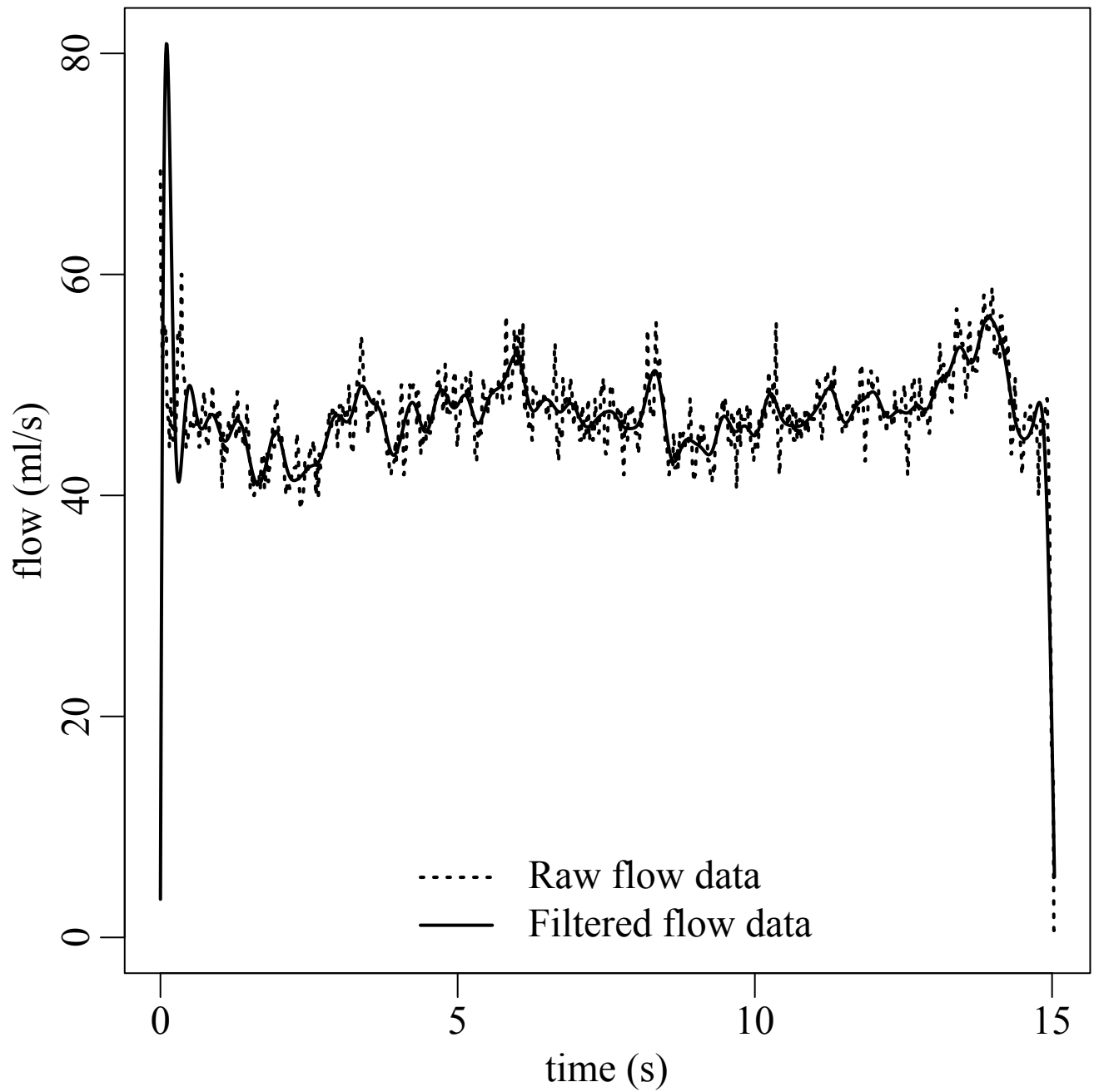

Supplement: Supplementary file 1 — Appendix S1. Supplemental information. [file PHY2-5-e13276-s001.zip › RawAndFilteredFlow.pdf]
